# Error in Figure Key

**DOI:** 10.1001/jamanetworkopen.2025.27228

**Published:** 2025-07-21

In the Research Letter titled “Location of Firearm Suicides in the United States, 2003-2021,”^1^ published June 9, 2025, the figure key stated “proportion at home” and it should have stated “proportion not at home.” This article has been corrected.^1^

## References

[1] Rencken C, Smart R, Ruben E, Rowhani-Rahbar A. Location of firearm suicides in the United States, 2003-2021. JAMA Netw Open. 2025;8(6):e2514423. doi:10.1001/jamanetworkopen.2025.14423 40489114 PMC12150191

